# Autophagy Is a Protective Response to the Oxidative Damage to Endplate Chondrocytes in Intervertebral Disc: Implications for the Treatment of Degenerative Lumbar Disc

**DOI:** 10.1155/2017/4041768

**Published:** 2017-02-22

**Authors:** Ke Chen, Xiaohua Lv, Wei Li, Fei Yu, Jianjing Lin, Junxuan Ma, Deming Xiao

**Affiliations:** ^1^Shenzhen Peking University-The Hong Kong University of Science and Technology Medical Center, Shenzhen, Guangdong 518036, China; ^2^Department of Pharmacology, Guangdong Medical University, Zhanjiang, Guangdong 524023, China; ^3^Department of Orthopaedics, Peking University Shenzhen Hospital, Guangdong 518036, China

## Abstract

Low back pain (LBP) is the leading cause of disability in the elderly. Intervertebral disc degeneration (IDD) was considered as the main cause for LBP. Degeneration of cartilaginous endplate was a crucial harmful factor during the initiation and development of IDD. Oxidative stress was implicated in IDD. However, the underlying molecular mechanism for the degeneration of cartilaginous endplate remains elusive. Herein, we found that oxidative stress could induce apoptosis and autophagy in endplate chondrocytes evidenced by western blot analysis, flow cytometry, immunofluorescence staining, GFP-LC3B transfection, and MDC staining. In addition, we also found that the apoptosis of endplate chondrocytes was significantly increased after the inhibition of autophagy by bafilomycin A1 shown by flow cytometry. Furthermore, mTOR pathway upstream autophagy was greatly suppressed suggested by western blot assay. In conclusion, our study strongly revealed that oxidative stress could increase autophagy and apoptosis of endplate chondrocytes in intervertebral disc. The increase of autophagy activity could prevent endplate chondrocytes from apoptosis. The autophagy in endplate chondrocytes induced by oxidative stress was mTOR dependent. These findings might shed some new lights on the mechanism for IDD and provide new strategies for the treatments of IDD.

## 1. Introduction

Low back pain (LBP) is the leading cause of disability in the elderly, resulting in low quality of life and high economic burden [1, 2]. About 70% of adults suffer from LBP at some points in their lifetime [3]. Intervertebral disc degeneration (IDD) was considered as the main cause for LBP [4, 5]. In addition, 95% individuals over age 50 had IDD in an autopsy investigation [6]. IDD, caused by genetic and environmental factors, is a multifactorial disease characterized by cellular and biochemical changes in disc tissue and progresses with age [7, 8].

Cartilaginous endplate (CEP) is a hydrated biological tissue that lies above and below the intervertebral disc, and CEP is the main route for the nutrition supply of intervertebral disc [9–12]. Emerging evidence showed that the degeneration of cartilaginous endplate, hindering the transport of nutrients, was a crucial harmful factor during the initiation and development of IDD [9–12]. However, the underlying molecular mechanism for the degeneration of cartilaginous endplate remains poorly understood, although it has been proved that excessive apoptosis of endplate chondrocytes was involved in this pathologic process [13–15].

Macroautophagy (autophagy), strongly associated with apoptosis, is an essential cellular self-eating process highly conserved in eukaryotic organism [16, 17]. Autophagy helps cells combat hostile situations through degrading unnecessary intracellular components, but excessive autophagy leads to cell death [18–20]. Recently, lines of study indicated that autophagy played an important role in the process of IDD [21–23]. Meanwhile, autophagy was also detected in endplate chondrocytes of intervertebral disc and proved to be implicated in the degeneration of cartilaginous endplate [24, 25].

Oxidative stress, resulting from overproduction of reactive oxygen species (ROS), was implicated in IDD by inducing premature senescence, promoting catabolic metabolism, and causing the apoptosis of intervertebral disc cells [26–29]. Another study revealed that the level of oxidative stress in the degeneration of cartilaginous endplate was increased, which indicated that oxidative stress was also involved in the degeneration of cartilaginous endplate [30]. Nonetheless, no study was designed to explore the autophagy and apoptosis of cartilaginous endplate under oxidative stress.

In the present study, we hypothesize that autophagy is a protective response to the oxidative damage to endplate chondrocytes in intervertebral disc. To prove our hypothesis, H_2_O_2_ was used to mimic oxidative stress. The change of autophagy and apoptosis of endplate chondrocytes together with the crosstalk between them was investigated.

## 2. Materials and Methods

### 2.1. Antibodies and Reagents

All cell culture reagents were from Gibco (CA, USA). 3-methyladenine (3MA), bafilomycin A1 (Baf), rapamycin, and monodansylcadaverine (MDC) were obtained from Sigma Aldrich (St. Louis, MO, USA). LC3B, p-mTOR, mTOR, pp70S6K, p70S6K, Bax, and Bcl-2 were purchased from Cell Signaling Technology (Beverly, MA, USA). H_2_O_2_ and toluidine blue were acquired from Sangon Biotech (Shanghai, China).

### 2.2. Isolation and Culture of Rat Endplate Chondrocytes

The authors' institutional Animal Care and Use Committee approved all the experimental protocol. Rat endplate chondrocytes were extracted using a method as described previously by Zhang et al. with minor revision [31]. Briefly, the cartilage endplate samples obtained from 4-week-old male Sprague-Dawley rats were minced into small pieces (1 mm^3^) (under a dissecting microscope with 4 magnifications) and digested with 0.25% trypsin for 30 min at 37°C. After being washed with PBS for three times, the samples were subject to 0.2% collagenase type II for 4 hours at 37°C. The isolated cells were cultured in complete culture medium (DMEM/F12) supplemented with 10% fetal bovine serum (FBS) and 1% penicillin/streptomycin. The second passage of primary endplate chondrocytes was used in the whole study.

### 2.3. HE Staining and Toluidine Blue

Cells seeded on sterile coverslips in 24-well plates were fixed in 4% paraformaldehyde at 4°C for 30 min. After being rinsed with PBS, cells were stained with hematoxylin and eosin (H&E) or 0.1% toluidine blue. Cells were washed extensively and photographed.

### 2.4. Immunofluorescence Staining

Cells were cultured on coverslips. After treatment with or without H_2_O_2_, cells were fixed with 4% paraformaldehyde for 30 min at 4°C. Cells were then subjected to 0.2% Triton X-100 for permeabilization at room temperature. Cells were blocked with 3% BSA for 30 min at room temperature followed by incubation with antibodies against LC3B (1 : 200) and type II collagen (1 : 200) overnight at 4°C. The next day, cells were washed with PBS and incubated with secondary antibodies conjugated by different fluorescence. Subsequently, Dapi was used to label the nuclei. Finally, images were detected by a fluorescence microscopy (Olympus, Japan).

### 2.5. Cell Viability Assay

Cells were seeded in 96-well plates. Viable cell numbers were detected by the use of Cell Counting Kit-8 (CCK-8, Dojindo, Kyushu, Japan) following the kit's instructions.

### 2.6. MDC

Monodansylcadaverine (MDC) is a specific marker for autolysosomes [32]. Cells were fixed in 4% paraformaldehyde for 30 min at 4°C. After being washed by PBS for three times, the cells were incubated with 0.2 mM MDC for 2 h at 37°C. Cells were then washed for four times and observed under a fluorescence microscope (Olympus, Japan).

### 2.7. GFP-LC3B Transfection

Cells were grown in 24-well plates with coverslips. Cells were transfected with the tandem GFP-RFP-LC3 adenovirus constructed by Hanbio Inc. (Shanghai, China). After 24 hours, cells were treated with or without 200 *μ*mol/L H_2_O_2_ for 2 hours. Subsequently, cells were fixed in 4% paraformaldehyde for 30 min at 4°C. Cells were subjected to a fluorescence microscope and the dots of GFP-LC3B were counted.

### 2.8. Western Blot

Cells were harvested and lysed by Western & IP Cell Lysis Kit (Beyotime, Jiangsu, China). Protein samples were separated by SDS-PAGE and transferred to nitrocellulose membranes. After being blocked with 5% nonfat milk at room temperature for 1.5 h, the membranes were incubated with primary antibodies against LC3B, mTOR, p-mTOR, p-p70S6K, p70S6K, Bax, Bcl-2, tubulin, and *β*-actin overnight at 4°C. Protein levels were quantified by Image J.

### 2.9. Apoptosis Detection by Flow Cytometry

Cells were prepared in 6-well plates. Apoptotic incidence was analyzed by the Annexin V-FITC/PI apoptosis detection kit (Life, USA) following the manufacturer's instructions. Briefly, cells were washed and harvested. Cells were then stained by Annexin V-FITC and PI (propidium iodide) for 15 min in the dark at room temperature. The cells were subjected to a flow cytometer (Beckman Coulter) within 1 h and apoptotic cells were quantified.

### 2.10. Statistical Analysis

Results were presented as means ± standard deviation. Statistical analysis was performed by SPSS 11.0 (SPSS, Chicago, IL, USA). One-way analysis of variance (ANOVA) was used for data analysis, followed by least significant difference test (Fisher test) and the unpaired Student's *t*-test was used for comparisons between two means. *p* values less than 0.05 were considered significant.

## 3. Results

### 3.1. The Identification of Endplate Chondrocytes from Lumbar Disc

**Figure 1:**
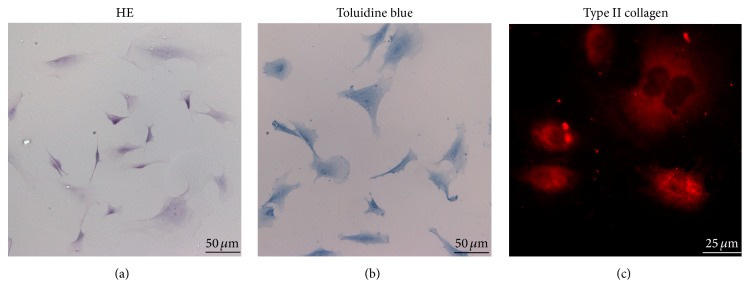
The identification of endplate chondrocytes. (a) Endplate chondrocytes were stained by HE. HE staining showed that majority of endplate chondrocytes were polygonal or spindle-shaped. (b) Endplate chondrocytes were labeled by toluidine blue. The cytoplasm of endplate chondrocytes exhibited blue after being labeled by toluidine blue. (c) Immunofluorescence staining for type II collagen. Type II collagen was distributed in the cytoplasm of endplate chondrocytes.

The results of HE staining verified that majority of endplate chondrocytes were polygonal or spindle-shaped (Figure 1(a)). The cytoplasm of endplate chondrocytes exhibited blue after being labeled by toluidine blue (Figure 1(b)). To further corroborate these findings, immunofluorescence staining for type II collagen (an important marker for chondrocytes) was carried out, and the data showed that type II collagen was distributed in the cytoplasm (Figure 1(c)).

All these data demonstrated that the cells harvested by us were endplate chondrocytes.

### 3.2. H_2_O_2_ Can Increase the Apoptosis of Endplate Chondrocytes

**Figure 2:**
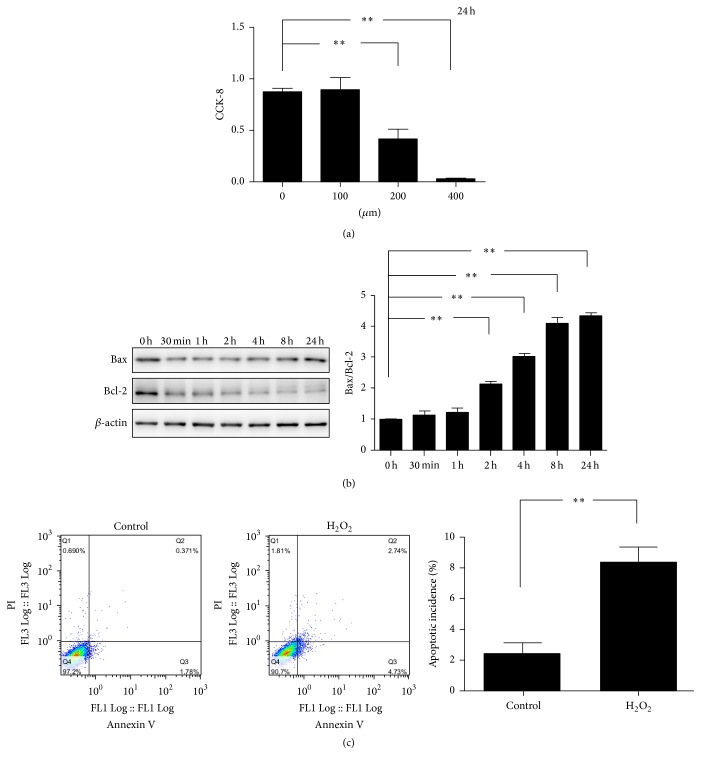
H_2_O_2_ increases the apoptosis of endplate chondrocytes. (a) Cell viability detected by CCK-8 assay. Endplate chondrocytes were treated with 0, 100, 200, and 400 *μ*M H_2_O_2_ for 24 h (*n* = 4). (b) The expression of Bax and Bcl-2 was examined by western blot after endplate chondrocytes were exposed to H_2_O_2_ (200 *μ*M) for different times (*n* = 3). (c) Apoptosis incidence of endplate chondrocytes was assessed by flow cytometry after treatment with or without 200 *μ*M H_2_O_2_ for 2 h (*n* = 3). The data are expressed as mean ± SD (^*∗*^*p* < 0.05; ^*∗∗*^*p* < 0.01).

To evaluate the effect of H_2_O_2_ on the survival of endplate chondrocytes, CCK-8 was used for the assay of cell viability. The results of CCK-8 showed that viable cells were significantly decreased after treatment with different concentrations of H_2_O_2_ for 24 h and 200 *μ*m/L of H_2_O_2_ had the definite cytotoxicity (Figure 2(a)). Thus, the concentration of 200 *μ*m/L of H_2_O_2_ was used in the following experiments.

To investigate the apoptosis response of endplate chondrocytes treated by oxidative stress, the protein expression of Bax and Bcl-2 was determined by western blot. As shown in Figure 2(b), a significant increase of Bax/Bcl-2 was observed in the endplate chondrocytes exposed to H_2_O_2_. Meanwhile, Annexin V and propidium iodide staining were also used to assess the apoptosis response of endplate chondrocytes under oxidative stress. As expected, H_2_O_2_ can greatly increase the apoptosis of endplate chondrocytes (Figure 2(c)).

Taken together, all these results suggested that oxidative stress can induce the apoptosis of endplate chondrocytes.

### 3.3. H_2_O_2_ Stimulates Autophagy in Endplate Chondrocytes

**Figure 3:**
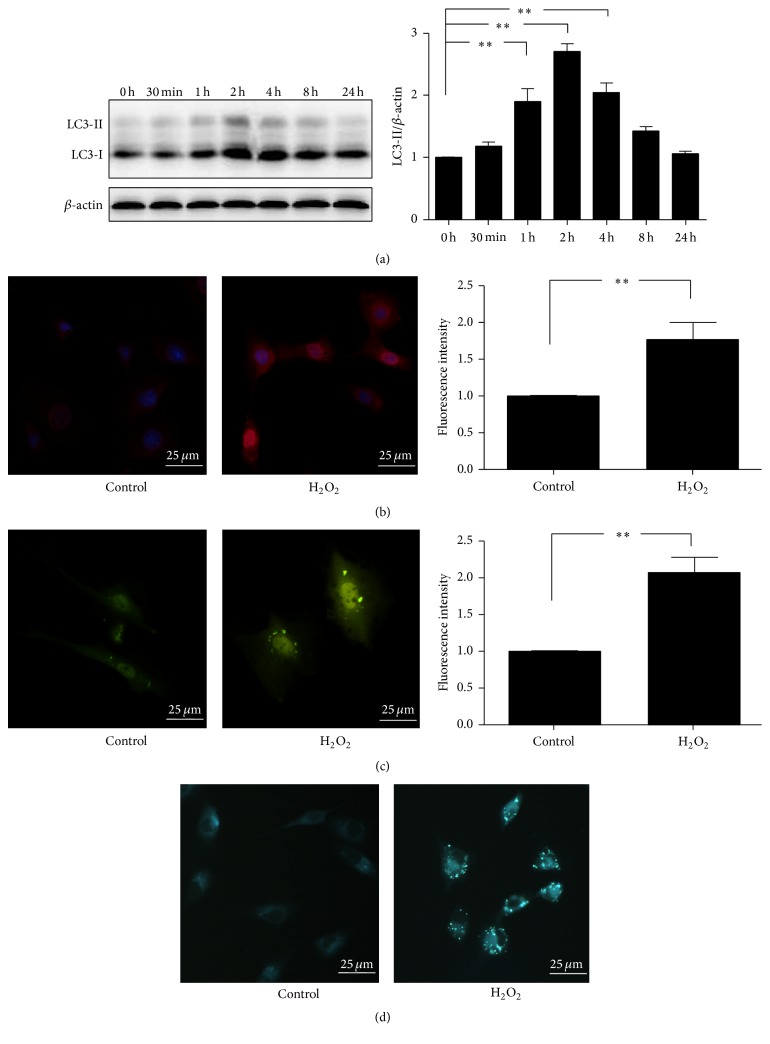
H_2_O_2_ stimulates autophagy in endplate chondrocytes. (a) The expression of LC3B-II in endplate chondrocytes was quantified by western blot after being stimulated by H_2_O_2_ for different times (*n* = 3). (b) Immunofluorescence assay for the expression of LC3B. Endplate chondrocytes were stained with LC3B after being exposed to H_2_O or H_2_O_2_ (200 *μ*M) for 2 h (*n* = 4). (c) GFP-LC3B in endplate chondrocytes was observed and quantified under a fluorescence microscope after treatment with or without 200 *μ*M H_2_O_2_ for 2 h (*n* = 5). (d) Autophagic vacuoles within endplate chondrocytes was labeled by MDC and detected by an fluorescence microscope after being exposure to 200 *μ*M H_2_O or H_2_O_2_ for 2 h. The data are expressed as mean ± SD (^*∗*^*p* < 0.05; ^*∗∗*^*p* < 0.01).

To investigate whether H_2_O_2_ can induce autophagy in endplate chondrocytes. Western blot was employed to detect the change of LC3B-II in endplate chondrocytes stimulated by 200 *μ*m/L of H_2_O_2_ at different times. The expression of LC3B-II reaches a peak at 2 h after being exposed to 200 *μ*m/L of H_2_O_2_ (Figure 3(a)) and 2 h was chosen as the time point in the next experiments.

In order to further confirm the results of western blot, immunofluorescence staining LC3B was performed. As shown in Figure 3(b), the expression of LC3B was significantly higher in the group treatment with H_2_O_2_ than that without H_2_O_2_.

In agreement with the LC3B evaluation, GFP-LC3B transfection also revealed that there were more GFP-LC3B dots in the cytoplasm of endplate chondrocytes treated by H_2_O_2_ compared with that treated by vehicle (Figure 3(c)).

The same tendency of higher autophagic activity stained by MDC was shown in the endplate chondrocytes stimulated by H_2_O_2_ (Figure 3(d)).

Altogether, these data indicated that H_2_O_2_ could activate autophagy in endplate chondrocytes.

### 3.4. Apoptosis of Endplate Chondrocytes Can Be Exacerbated by H_2_O_2_ after the Inhibition of Autophagy

**Figure 4:**
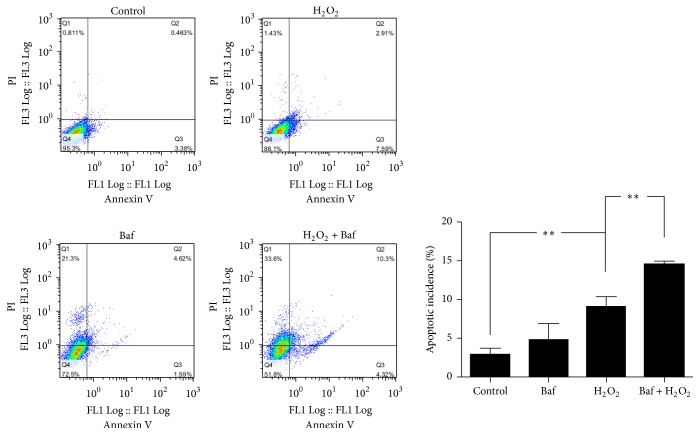
Apoptosis of endplate chondrocytes can be exacerbated by H_2_O_2_ after the inhibition of autophagy. Apoptosis incidence of endplate chondrocytes was assessed by flow cytometry after treatment with H_2_O, H_2_O_2_ (200 *μ*M), bafilomycin A1 (Baf, 100 nM), H_2_O_2_, and Baf + H_2_O_2_ for 24 h (*n* = 3). The data are expressed as mean ± SD (^*∗*^*p* < 0.05; ^*∗∗*^*p* < 0.01).

Since autophagy is a protective response to the apoptosis of osteoblasts in previous studies, flow cytometry was exerted to validate whether autophagy is also a prosurvival reaction to the apoptosis of endplate chondrocytes under oxidative stress [33] (Figure 4). Strikingly, when the endplate chondrocytes were subjected to H_2_O_2_, the apoptosis incidence was drastically enhanced after the inhibition of autophagy by bafilomycin A1 (Figure 4). By contrast, bafilomycin A1 alone could not increase the apoptosis of endplate chondrocytes (Figure 4).

These data revealed that autophagy was a protective response to the apoptosis of endplate chondrocytes under oxidative stress.

### 3.5. H_2_O_2_ Induces Autophagy through the mTOR Pathway in Endplate Chondrocytes

**Figure 5:**
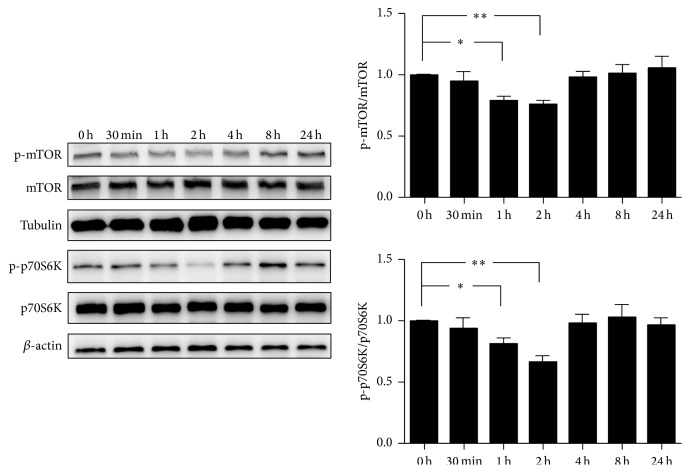
H_2_O_2_ induces autophagy through the mTOR pathway in endplate chondrocytes. The phosphorylation of mTOR and p70S6K was evaluated by western blot after being treated with 200 *μ*M H_2_O_2_ at different times (*n* = 3). The data are expressed as mean ± SD (^*∗*^*p* < 0.05; ^*∗∗*^*p* < 0.01).

It has been reported that mTOR is an important modulator of autophagy and the inhibition of phospho-mTOR can trigger autophagy [33]. To clarify the phosphorylation of mTOR pathway in endplate chondrocytes treated by H_2_O_2_. Western blot was employed to detect the protein expressions of p-mTOR and p-p70S6K. The results indicated that the expressions of p-mTOR and p-p70S6K were greatly downregulated in endplate chondrocytes after being activated by H_2_O_2_ (Figure 5).

These evidence suggested that the autophagy induced by H_2_O_2_ is mTOR dependent.

## 4. Discussion

IDD is a major cause of LBP. Currently, the standard therapeutic strategy for IDD includes physical therapy, anti-inflammatory medications, and surgical treatment [34]. As we know, surgical operations have detrimental complications such as recurrence, degeneration of adjacent segment, and change of mechanical properties [35, 36]. Therefore, biological therapy is a potential method for the treatment of IDD, which can avoid the complication of surgery. Numerous researches have shown that oxidative stress participated in the pathological process of IDD [26–30, 37]. However, the underpinning molecular mechanism is only partially elucidated.

In this study, we found that both autophagy and apoptosis in endplate chondrocytes were elevated after being exposed to H_2_O_2_ and the apoptosis was dramatically enhanced when autophagy was repressed, suggesting that autophagy plays a protective response to apoptosis of endplate chondrocytes under the stimulation of H_2_O_2_. Meanwhile, we also observed that the phosphorylation of mTOR and p70S6K was suppressed by H_2_O_2_, indicating that mTOR pathway was implicated in the activation of autophagy when the endplate chondrocytes were subjected to H_2_O_2_.

It was reported that H_2_O_2_ can induce autophagy in many kinds of cells [38–40]. Meanwhile, some reports showed that H_2_O_2_ could increase autophagy in nucleus pulposus cells of rats [41]. Our finding was inconsistent with previous studies but the concentration of H_2_O_2_ used by us was different from that in other experiments [38–41]. In contrast, other reports revealed that H_2_O_2_ blocks rather than induces autophagy in some cells [42]. One possible reason was that different kinds of cells had different reactions under the same stimulation of oxidative stress.

H_2_O_2_ can increase the apoptosis incidence of endplate chondrocytes. The increase of apoptosis incidence caused by H_2_O_2_ was also observed in NP cells and osteoblasts [38, 41]. Our present data concurs with previous discoveries, in which H_2_O_2_ could also aggravate the apoptosis incidence of nucleus pulposus cells and osteoblasts [38, 41].

The relationship between autophagy and apoptosis is complicated. It has been recognized that appropriate autophagy can help cells against apoptosis or, alternatively, excessive autophagy can lead to death [43, 44]. In order to clarify the effect of autophagy in endplate chondrocytes under the stimulation of H_2_O_2_, we applied bafilomycin A1 to block the autophagy. Notably, the apoptosis of endplate chondrocytes was significantly enhanced after the inhibition of autophagy. Our results were in line with reports that state that the role of autophagy is a protective response to cytotoxic stimuli [21, 38]. However, data from other studies were contradictory to ours, which showed that autophagy was a prodeath response during oxidative stress [41, 45]. Presumably, the prosurvival or prodeath effect of autophagy under oxidative stress might be dependent on cell type, cell environment, basal autophagy activity, and time or degree of stimulation. Autophagy in endplate chondrocytes was an early reaction to H_2_O_2_, which could reduce the generation of intracellular ROS and facilitate the survival of endplate chondrocytes being exposed to oxidative stress, whereas excessive autophagy activated by H_2_O_2_ destroyed lots of cellular components and made the cell fail to survival.

mTOR pathway was reported to be an important modulator upstream autophagy [46]. Other researches demonstrated that autophagy can also be activated independent of mTOR [16]. Thus, western blot was used to evaluate the phosphorylation of mTOR and p70S6K. Interestingly, both the phosphorylation of mTOR and p70S6K in endplate chondrocytes were impeded under oxidative stress. Our results revealed that H_2_O_2_-mediated autophagy was mTOR dependent. This result was in accordance with previous studies [38, 41].

Several limits exist in our present experiments. Firstly, data obtained in vitro may not be the same as data in vivo. Therefore, experiments in vivo should be done to further evaluate the results acquired from in vitro. Secondly, more mechanisms such as MAPK pathway upstream mTOR should be investigated. Thirdly, gene silencing might be a better way to inhibit autophagy instead of chemical modulators which was used in this study.

In summary, our results support that H_2_O_2_ can induce autophagy and apoptosis in endplate chondrocytes of rats. Furthermore, autophagy is a protective response to the apoptosis elevated by H_2_O_2_. The autophagy induced by H_2_O_2_ is mTOR pathway. Our findings revealed that the regulation of autophagy may be helpful in developing strategies to suppress IDD.

## Additional Points

*Highlights*. Oxidative stress increases autophagy and apoptosis of endplate chondrocytes. Autophagy is a protective response to the oxidative damage to endplate chondrocytes. Autophagy induced by oxidative stress is mTOR dependent.
